# No Evidence for a Decrease in Physical Activity Among Swiss Office Workers During COVID-19: A Longitudinal Study

**DOI:** 10.3389/fpsyg.2021.620307

**Published:** 2021-02-11

**Authors:** Andrea Martina Aegerter, Manja Deforth, Gisela Sjøgaard, Venerina Johnston, Thomas Volken, Hannu Luomajoki, Julia Dratva, Holger Dressel, Oliver Distler, Markus Melloh, Achim Elfering, the NEXpro Collaboration Group, Andrea M. Aegerter

**Affiliations:** ^1^Institute of Health Sciences, School of Health Professions, Zurich University of Applied Sciences, Winterthur, Switzerland; ^2^Department of Sports Science and Clinical Biomechanics, University of Southern Denmark, Odense, Denmark; ^3^School of Health and Rehabilitation Sciences, The University of Queensland, Brisbane, QLD, Australia; ^4^Institute of Physiotherapy, School of Health Professions, Zurich University of Applied Sciences, Winterthur, Switzerland; ^5^Faculty of Medicine, University of Basel, Basel, Switzerland; ^6^Division of Occupational and Environmental Medicine, Institute of Epidemiology, Biostatistics and Prevention, University Hospital Zurich, University of Zurich, Zurich, Switzerland; ^7^Department of Rheumatology, University Hospital Zurich, University of Zurich, Zurich, Switzerland; ^8^School of Medicine, The University of Western Australia, Perth, WA, Australia; ^9^Curtin Medical School, Curtin University, Bentley, WA, Australia; ^10^Institute of Psychology, University of Bern, Bern, Switzerland

**Keywords:** coronavirus, COVID-19, SARS-CoV-2, lockdown, physical exercise, health promotion, public health, shutdown

## Abstract

**Purpose:**

The COVID-19 lockdown interrupted normal daily activities, which may have led to an increase in sedentary behavior (Castelnuovo et al., 2020). The aim of this study was to investigate the effect of the COVID-19 pandemic on the level of physical activity among Swiss office workers.

**Methods:**

Office workers from two Swiss organizations, aged 18–65 years, were included. Baseline data from January 2020 before the COVID-19 pandemic became effective in Switzerland were compared with follow-up data during the lockdown phase in April 2020. Levels of physical activity were assessed using the International Physical Activity Questionnaire. Paired sample *t*-tests or Wilcoxon signed-rank test were performed for statistical analysis.

**Results:**

Data from 76 participants were analyzed. Fifty-four participants were female (71.1%). The mean age was 42.7 years (range from 21.8 to 62.7) at baseline. About 75% of the participants met the recommendations on minimal physical activity, both before the COVID-19 pandemic and during the lockdown. Weak statistical evidence for a decline in total physical activity in metabolic equivalent of task minutes per week (MET min/week) was found (estimate = −292, 95% CI from – ∞ to 74, *p*-value = 0.09), with no evidence for a decrease in the three types of activity: walking (estimate = −189, 95% CI from – ∞ to 100, *p*-value = 0.28), moderate-intensity activity (estimate = −200, 95% CI from – ∞ to 30, *p*-value = 0.22) and vigorous-intensity activity (estimate = 80, 95% CI from – ∞ to 460, *p*-value = 0.74). Across the three categories “high,” “moderate,” and “low” physical activity, 17% of the participants became less active during the lockdown while 29% became more active.

**Conclusion:**

The COVID-19 pandemic did not result in a reduction in total physical activity levels among a sample of Swiss office workers during the first weeks of lockdown. Improved work-life balance and working times may have contributed to this finding.

**Clinical Trial Registration:**

www.ClinicalTrials.gov, NCT04169646. Registered 15 November 2019 – Retrospectively registered, https://clinicaltrials.gov/ct2/show/NCT04169646.

## Introduction

Following the declaration of coronavirus disease (COVID-19) as a global pandemic by the World Health Organization on 11th of March 2020, many countries worldwide have enforced a societal-level lockdown. In Switzerland, the lockdown began 5 days later. Stores, schools, colleges, and sports facilities were temporarily closed, office workers were advised to work from home, and the public was recommended to stay at home if possible. Open spaces and green areas, however, remained open as long as the social distancing rules were respected. In comparison to other countries, the lockdown in Switzerland can be described as soft, as a curfew or restrictions to movement outside of the house were never imposed.

Nevertheless, the national restrictions interrupted normal daily activities, especially physical activity, and people spent more time at home, often lying down or sitting (Chandrasekaran and Ganesan, 2020). This may result in an increase in sedentary behavior, which is considered a major risk factor for the development or worsening of chronic diseases such as obesity, cancer, and cardiovascular diseases (Gibbs et al., 2015). Moreover, these three chronic diseases are among the leading causes of deaths worldwide (World health statistics, 2020) and are all risk factors for the development of a more severe COVID-19 outcome (Zheng et al., 2020). In addition, the level of physical activity is associated with the risk of a community-acquired pneumonia in women, with pneumonia being a major complication of COVID-19 (Baik et al., 2000). It is also known that greater physical activity is associated with a lower risk of musculoskeletal pain (Kirsch Micheletti et al., 2019) and improves mental health issues, such as mood and depression, which is particularly relevant during social isolation (Hammig et al., 2011; Cooney et al., 2013; McDowell et al., 2019; Denay et al., 2020; Meyer et al., 2020). Physical activity in fact has been shown to be similarly effective as psychological therapy and drug therapy in depression (Cooney et al., 2013). Furthermore, being physically active is associated with a 22% reduced risk of becoming depressed and having a better mood than being physically inactive (Hammig et al., 2011). A strong positive association was also found between physical activity and reaction time and memory (Magnon et al., 2018). In contrast, anxiety had a negative influence on the intention to be physically active (Chirico et al., 2020).

The known benefits of exercise have prompted several research groups to investigate the topic of physical activity and COVID-19. A study conducted in Spain demonstrated that the number of participants who followed the World Health Organization recommendations on levels of physical activity decreased from 60.6% before the lockdown to 48.9% in the first week of isolation (López-Bueno et al., 2020). In another study, 75% of participants met the physical activity guidelines during the lockdown, with women achieving higher values than men (Smith et al., 2020). A Belgian study came to a similar conclusion and showed that physically active adults, who normally exercised in a group setting and did not use online training tools during social distancing, were less active than before (Constandt et al., 2020). The same applied to many patients with type 2 diabetes mellitus, heart failure, cystic fibrosis, or neuromuscular diseases, who experienced increasing physical inactivity during home confinement (Di Stefano et al., 2020; Radtke et al., 2020; Ruiz-Roso et al., 2020; Vetrovsky et al., 2020). Two large surveys also showed that the lockdown had a negative effect on physical activity levels (Ammar et al., 2020; Qin et al., 2020). In contrast, one study reported that adults who were physically less active before the lockdown were more active during the lockdown (Constandt et al., 2020). An analysis of Google’s relative search rates also showed that the general population’s interest in physical activity increased during the lockdown (Ding et al., 2020).

Previous literature on physical activity and COVID-19 has mainly focused on the general population, frail individuals (elderly or sick), health care professionals or athletes. Office workers are clearly underrepresented, although they accounted for about 13% of the Swiss workforce (600,000 office workers) during the lockdown (Bundesamt für Statistik (BFS), 2020), so investigating this sample seems highly relevant. Further, a common limitation of the existing literature is that data collection did not commence until the outbreak of COVID-19, which is subject to recall bias, and that the changes in physical activity were sparsely quantified. In the present study, baseline data were collected before the COVID-19 pandemic and a validated tool was used to enable quantification of the physical activity levels and comparison with international guidelines. The aim of this analysis was to quantify the effect of the COVID-19 pandemic on physical activity levels among Swiss office workers. Considering the current literature, we hypothesized that total physical activity decreased during the first few weeks of the COVID-19 lockdown phase.

## Materials and Methods

### Design and Participants

**FIGURE 1:**
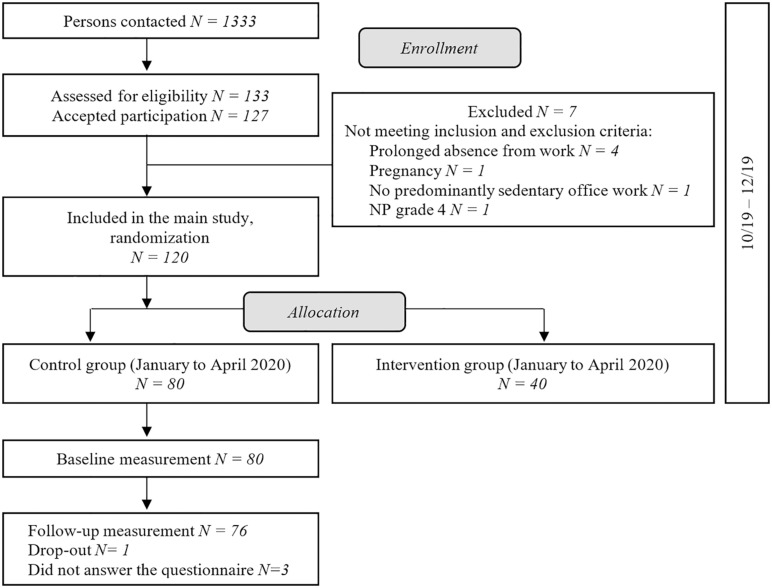
Study flow-chart.

This is a longitudinal study based on data from an ongoing randomized controlled trial (Aegerter et al., 2020). The study was approved by the Ethics Committee of the Canton of Zurich, Switzerland (swissethics no. 2019-01678). Participants were recruited between October and December 2019 from two Swiss organizations in the Cantons of Zurich and Aargau. Inclusion criteria were Swiss office workers aged 18–65 years, working more than 25 h per week (0.6 full-time equivalent) in predominantly sedentary office work, able to communicate in German (written, spoken), and provided written informed consent (Aegerter et al., 2020). Exclusion criteria were severe health conditions such as previous trauma or injuries of the neck, inflammatory disease, any history of cervical spine surgery, pregnancy or if exercise was contraindicated (Aegerter et al., 2020). For this analysis, only participants in the control cohort (similar to a waiting list) between January and April 2020 were included (Figure 1).

### Procedure

Baseline data used in this analysis is the level of physical activity 10 weeks before the COVID-19 pandemic became effective (first case) in Switzerland (January 2020), while the follow-up data represent the physical activity during the fourth (and fifth) week of lockdown (April 2020). All data were collected through a 30-min online questionnaire, but only the International Physical Activity Questionnaire: short last 7 days self-administered format – German Version (IPAQ-SF) data were analyzed for this study. UNIPARK^©^ (Berlin, Germany) was the platform used to host the online questionnaire. Participants without follow-up measures were excluded from the analysis (*n* = 4/80). The STROBE Statement checklist was used for the reporting of this study (von Elm et al., 2007).

### Outcomes and Measures

#### Physical Activity

Physical activity was assessed using the IPAQ-SF. The IPAQ-SF is a validated, reliable (Craig et al., 2003), and cost-effective method for assessing physical activity (Lee et al., 2011). The IPAQ-SF asks for physical activity (e.g., during leisure time, housework, gardening, work, and transportation) within the last 7 days, and distinguishes three activity types (walking, moderate-intensity activity, and vigorous-intensity activity). Data processing and analysis were performed according to the IPAQ-SF guidelines (IPAQ group, 2020).

##### Physical activity as a continuous measure

Metabolic equivalent of task minutes per week (MET min/week) was calculated for the three types of activity (walking = 3.3 × walking minutes × walking days; moderate-intensity activity = 4.0 × moderate-intensity activity minutes × moderate days; vigorous-intensity activity = 8.0 × vigorous-intensity activity minutes × vigorous-intensity days), whereas the sum of the three types equaled the total physical activity level (primary outcome). According to the IPAQ-SF guidelines, participants who indicated “not sure” about the number of physically active minutes in an activity type were considered as “missing” in the corresponding type of activity, but included in the total physical activity calculation (IPAQ group, 2020). There are no studies available on the minimal clinically important difference in MET min/week of the IPAQ-SF.

##### Physical activity categories

Based on their weekly physical activity level, participants were classified into one of the three categories “low,” “moderate,” and “high” (Pedersen et al., 2009; U.S. Department of Health and Human Services, 2018). The category “high” was assigned if participants performed 3 days or more of vigorous-intensity activity achieving a minimum total physical activity of at least 1,500 MET-min/week or 7 days of any combination achieving a minimum total physical activity of at least 3,000 MET-min/week (IPAQ group, 2020). Criteria for the category “moderate” were: 3 days or more of vigorous-intensity activity of at least 20 min per day; 5 days or more of moderate-intensity activity and/or walking of at least 30 min per day; or 5 days or more of any combination achieving a minimum total physical activity of at least 600 MET-min/week (IPAQ group, 2020). Participants who could not be categorized as “high” or “moderate” were classified as “low” (IPAQ group, 2020). Only the categories “high” and “moderate” meet the recommendations on minimal physical activity of the U.S. Department of Health and Human Services and the World Health Organization (Pedersen et al., 2009; World Health Organization, 2010; U.S. Department of Health and Human Services, 2018).

#### Participants’ Characteristics

Data on participants’ age, gender, and body-mass-index (BMI) were obtained. At follow-up, participants rated their work-life balance and their working times (including start of work, end of work, work duration, and breaks) on a numeric rating scale (NRS) with a score from 1 (clearly better than before the COVID-19 pandemic) to 5 (clearly worse than before the COVID-19 pandemic).

### Statistical Analysis

Participants’ characteristics were analyzed using descriptive statistics with mean or median values (including standard deviation or quartiles), minimum and maximum value or, in the case of factor variables, with absolute and relative frequencies.

The normality assumption was investigated by Q-Q plots, boxplots, and Shapiro–Wilk test. To test the mean difference in total physical activity (MET min/week) between baseline and follow-up measures, one-sided paired sample *t*-test was used. If the assumption of normal distribution was not met, Wilcoxon signed-rank test was performed. The same procedure was applied for the analysis of each of the three types of physical activity (walking, moderate-intensity activity, and vigorous-intensity activity), including Bonferroni-Holm correction for multiple comparisons. In addition, an exploratory graphical analysis of the effect of BMI and gender on the MET min/weeks was conducted.

For the categorical analysis of physical activity, absolute frequencies of participants classified into the categories “low,” “moderate,” and “high” physical activity at baseline and follow-up were calculated. Changes in categories between baseline and follow-up measurement were presented graphically by a mosaic plot. The percentage of participants meeting the recommendations for minimal physical activity level, which means a classification into “high” or “moderate” physical activity category, was evaluated for baseline and follow-up.

All analyses were conducted in R Project for Statistical Computing (R Core Team, 2020), version 4.0.2, using the base packages and the following analysis-specific packages: beeswarm, car, dplyr, ggplot2, lubridate, tableone, vcd. Significance level alpha was set at 0.05. The *p*-values were expressed as the strength of evidence with very strong evidence (*p* ≤ 0.001), strong evidence (0.001 < *p* ≤ 0.01), evidence (0.01 < *p* ≤ 0.05), weak evidence (0.05 < *p* ≤ 0.1), and little or no evidence (*p* > 0.1) (Bland, 2015). The data analyst was blinded to the identity of the participants.

## Results

### Participants

Four participants were excluded including one who withdrew and three who did not complete the online questionnaire at follow-up, resulting in 76 participants remaining for the analysis (Figure 1). The average time between completion of the questionnaire at the two time points was 102 days (±9 days).

The mean age of the participants was 42.7 years (range: 21.8 to 62.7 years) at baseline. About 70% of the participants were female (*n* = 54). The average BMI was 23.9 kg/m^2^ (±3.5 kg/m^2^) at baseline and 23.7 kg/m^2^ at follow-up (±3.5 kg/m^2^). Approximately, 80% (*n* = 60) of participants had Swiss nationality. Seventy-six percent (*n* = 58) of the participants had a tertiary level education, 22% (*n* = 17) completed upper secondary education and 1.3% (*n* = 1) primary compulsory education. There was no statistical evidence for a difference in participant’s characteristics between baseline and follow-up.

A better work-life balance during the lockdown than before the COVID-19 pandemic was reported by 43.4% of participants (*n* = 33), while it remained unchanged in 28.9% (*n* = 22) and worsened in 22.4% of participants (*n* = 17; missing values in 5.3%, *n* = 4). Similarly, working times were rated to be better by 38.2% of the participants (*n* = 29), unchanged by 21.1% (*n* = 16) and worsened by 35.5% of the participants (*n* = 27; missing values in 5.3%, *n* = 4).

### Continuous Measure of Physical Activity

**FIGURE 2:**
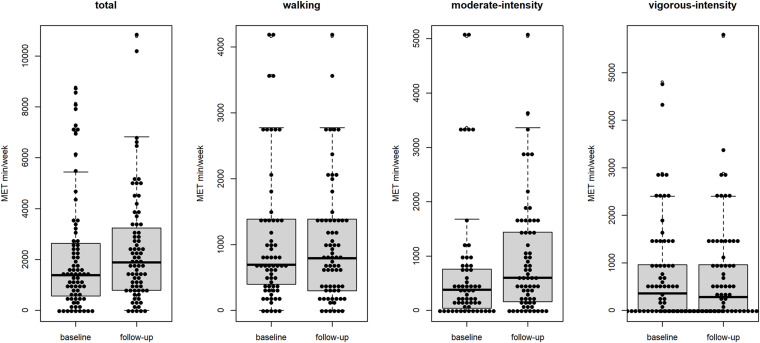
Physical activity in MET min/week at baseline (before the COVID-19 pandemic) and follow-up (during the lockdown).

The descriptive statistics of the outcomes at baseline and follow-up are shown in Table 1. The assumption of the data being normally distributed was met for the physical activity type of walking (*p*-value = 0.21), but not for the types of activity: moderate-intensity activity, vigorous-intensity activity, and total physical activity (all with a *p*-value < 0.05). There was only weak evidence for a decline in total physical activity measured by MET min/week between baseline and follow-up (estimate = −292, 95% CI from – ∞ to 74, *p*-value = 0.09). However, no evidence was found for a decline in the three types of activity: vigorous-intensity activity (estimate = 80, 95% CI from – ∞ to 460, *p*-value = 0.74), moderate-intensity activity (estimate = −200, 95% CI from – ∞ to 30, *p*-value = 0.22), and walking (estimate = −189, 95% CI from – ∞ to 100, *p*-value = 0.28) between both measurements (Figure 2). An explorative graphical analysis showed no effect of BMI and gender on the result (Supplementary Material).

**TABLE 1:** Participant characteristics at baseline (before the COVID-19 pandemic) and follow-up (during the lockdown).

	Baseline (*N* = 76)	Follow-up (*N* = 76)
**Age [years]**		
Mean (SD)	42.7 (9.2)	
Median (Min, Max)	42.2 (21.8, 62.7)	
**Gender**		
Female (%)	54 (71.1)	
Male (%)	22 (28.9)	
**Nationality**		
Swiss (%)	60 (78.9)	
Other (%)	16 (21.1)	
**Education**		
Tertiary level education (%)	58 (76.3)	
Upper secondary education (%)	17 (22.4)	
Primary compulsory Education (%)	1 (1.3)	
**Body-Mass-Index (BMI)**		
Mean (SD)	23.9 (3.5)	23.7 (3.5)
**Total physical activity [MET minutes/week]**		
Mean (SD)	2150 (2310)	2370 (2150)
Median (Min, Max)	1390 (0, 8760)	1890 (0, 10800)
**Vigorous-intensity activity [MET minutes/week]**		
Mean (SD)	749 (1060)	705 (1050)
Median (Min, Max)	360 (0, 4800)	280 (0, 5760)
Missing	5 (6.6%)	6 (7.9%)
**Moderate-intensity activity [MET minutes/week]**		
Mean (SD)	727 (1160)	929 (1020)
Median (Min, Max)	380 (0, 5040)	600 (0, 5040)
Missing	16 (21.1%)	10 (13.2%)
**Walking [MET minutes/week]**		
Mean (SD)	1030 (1030)	981 (890)
Median (Min, Max)	693 (0, 4160)	792 (0, 4160)
Missing	11 (14.5%)	5 (6.6%)

*Max, maximum; MET, metabolic equivalent of task; Min, minimum; SD, standard deviation.*

### Categorical Measure of Physical Activity

**FIGURE 3:**
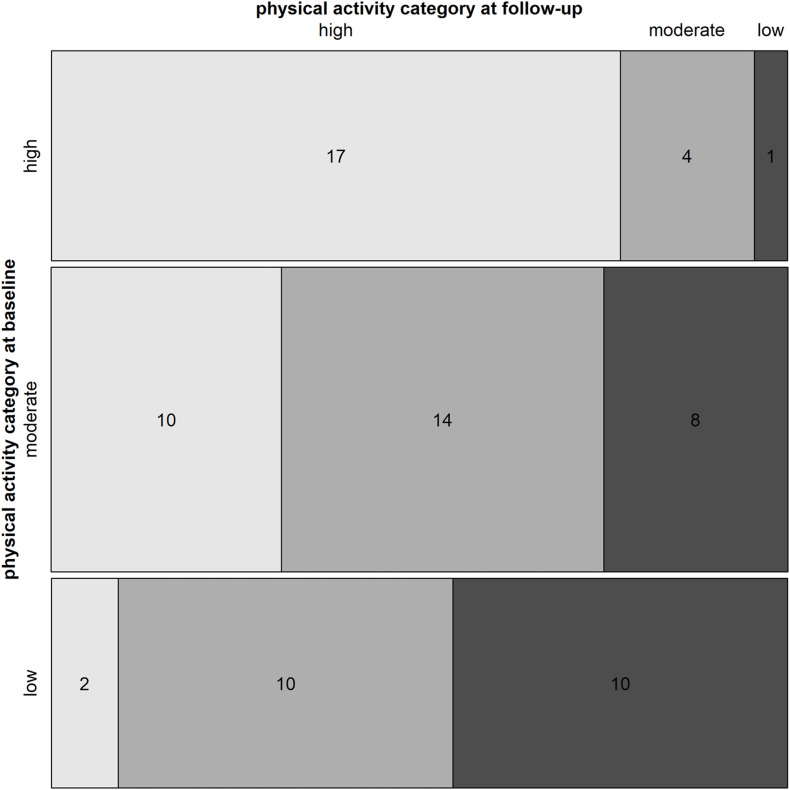
Physical activity categories at baseline (before the COVID-19 pandemic) and follow-up (during the lockdown). The mosaic plot shows three horizontal bars, with one bar per category of physical activity (“low,” “moderate,” and “high”). The colored areas of the horizontal bars represent the proportion of the study participants which reached the physical activity categories “high” (light gray), “moderate” (gray), and “low” (dark gray) at follow-up. The numbers represent the number of participants.

The mosaic plot in Figure 3 shows the classification into the three physical activity categories (“high,” “moderate,” and “low”) at baseline and follow-up as well as the change in category between these two points in time. At baseline, 29% (*n* = 22) of participants were classified as “high,” 42% (*n* = 32) as “moderate,” and 29% (*n* = 22) as “low.” Among the participants at baseline being classified as “high,” 77% (*n* = 17) remained in the same category during the follow-up. Correspondingly, 75% among those classified as “moderate” remained in the same (44%, *n* = 14) or advanced to the “high” (31%, *n* = 10) category at follow-up. In the category “low,” 55% of the participants (*n* = 12) increased to a higher category at follow-up. Furthermore, the mosaic plot shows that 71% of participants (*n* = 54) fulfilled the recommendations on minimal physical activity level at baseline, compared to 75% of the participants (*n* = 57) at follow-up.

## Discussion

### Summary of Findings

About three-quarter of the study participants met the recommendations for minimal physical activity before the COVID-19 pandemic. This high percentage remained unchanged during the COVID-19 lockdown. Across the three physical activity categories of “high,” “moderate,” and “low,” about 17% of the participants became less active during the lockdown phase, 54% of participants maintained their physical activity level, and an increase was recorded in 29%. Regarding the primary outcome of total physical activity in MET min/week, our hypothesis was rejected, i.e., total physical activity did not decline during the first weeks of the COVID-19 lockdown. Similarly, there was no evidence of a decline in any of the three types of physical activity (walking, moderate-intensity activity, and vigorous-intensity activity). The majority of participants rated their work-life balance (72.2%) and the working times (59.3%) as better or unchanged during the lockdown compared to before the COVID-19 pandemic.

### Interpretation and Comparison With Literature

Our primary outcome of total physical activity and the three types of physical activity (walking, moderate-intensity activity, and vigorous-intensity activity), all of which are expressed as MET min/week, showed no evidence for a statistical significant difference under the lockdown compared to the situation before the COVID-19 pandemic. This result contrasts with the results of two large international surveys (Ammar et al., 2020; Qin et al., 2020), which showed that the lockdown had a negative effect on physical activity levels. However, both surveys had a risk of recall bias and no baseline measurement was performed before the COVID-19 pandemic. Nevertheless, the question arises for this unexpected result with several possible explanations. First, the baseline data collected in January 2020 could have been artificially increased or decreased due to New Year’s resolution, winter breaks in sports clubs or bad weather conditions (e.g., no cycling to work or jogging because of snow). Second, the follow-up data could also be affected positively or negatively, for example through the individual fear of an infection (e.g., risk group versus non-risk group), working from home (e.g., no way to work), fewer low-intensity activities (e.g., shopping), the good weather during the lockdown [(MeteoSchweiz, 2020), e.g., spending more time outside compared to January], the increased leisure time or changed working hours. The latter two reasons can be supported by the results of our study, which show that work-life balance and working times seem to be better during the lockdown. Third, the main motives for doing sports are “for my health” and “to be fit” (Lamprecht et al., 2020). Therefore, it is possible that the participants consciously were more physically active during the lockdown in order to compensate for the loss in their freedom to move around. Fourth, not enough time may have elapsed since initiation of the lockdown to see a real change in physical activity levels. It may be that office workers will compensate for their physical activity level with different strategies over the time course of the pandemic. Fifth, and probably most important, countries like Spain had stricter lockdown measures, such as a ban on physical activity outside the home, which could explain the different results.

The recently published Swiss Health Survey showed that 75% of Swiss respondents followed the recommendations for minimal physical activity before the COVID-19 pandemic, which is supported by our finding of 71% (Lamprecht et al., 2020). Compared to other countries, such as Spain, the percentage of participants meeting the recommendations before the COVID-19 pandemic is 15% higher in Switzerland (López-Bueno et al., 2020). A similar finding was made by Ting et al. (2019), who reported that only 66% of Australian office workers were sufficiently physically active to promote health before the COVID-19 pandemic. This indicates that the country and the culture are important determinants of physical activity levels. Other findings of interest might be that, in contrast to a pilot study from Turkey, it could not be confirmed that men were more physically active than women (Tek et al., 2020). The Swiss Health Survey also found no gender-specific differences in physical activity, but for region, nationality, age and education level (Lamprecht et al., 2020). More physical activity is performed in the German-speaking regions and by Swiss nationals compared to foreigners living in Switzerland (Lamprecht et al., 2020). Furthermore, physical activity reductions were found with age and lower education level (Lamprecht et al., 2020).

The percentage of participants who met the recommendations for minimal physical activity during the lockdown phase is in line with the results of a study on United Kingdom adults, both achieving 75% (Smith et al., 2020). This can be explained by the fact that the governmental restrictions and the COVID-19’s spread were quite similar in both countries. Again in Spain, where the regulations were very restrictive, a considerable decline below 50% of participants meeting the minimal physical activity recommendations during the lockdown was found (López-Bueno et al., 2020). In contrast, a study among Canadians found that 22.4% of physically active participants who performed at least 150 min of moderate-vigorous physical activity per week became less active during the lockdown (Lesser and Nienhuis, 2020). Using the definition of “physically active” as participants in the category “high” or “moderate,” our study achieves a very similar result with 24% (*n* = 13 out of 54) of participants who became less active during the lockdown. This reduction in physical activity could be explained by the fact that moderately or highly physically active participants usually performed their exercises in training groups, fitness centers, and sports clubs, that were temporarily closed during the lockdown (Constandt et al., 2020). Further reasons could be less time, the lack of a competitive element in the training or being at risk for developing COVID-19 (Constandt et al., 2020; Di Stefano et al., 2020; Ruiz-Roso et al., 2020; Vetrovsky et al., 2020). Interestingly, 33% of inactive participants were reported to be more active during the lockdown in Canada (Lesser and Nienhuis, 2020), while this was the case for as many as 55% (*n* = 12 out of 22) in our study on Swiss office workers. This result is also in line with the findings of a Belgian study, which argued that the health benefits of training seemed important enough to motivate less active people to increase their activity level (Constandt et al., 2020).

Another point to discuss is whether the mean value of the total physical activity, which was in our case 2,150 MET min/week at baseline and 2,370 MET min/week at follow-up, is generally considered high or low. One of the main reasons for this is that there are no official reference values. Further difficulties in comparison may arise from the fact that some studies included other MET values in their calculations, e.g., values between four and six MET for moderate-intensity activity (Nelson et al., 2007), compared to three MET used in our calculation and in the official IPAQ-SF guidelines. In consequence comparability is limited. As an example, a study on university students yielded total physical activity of 5,373 MET min/week, which is more than twice as high as in the present study (Fagaras et al., 2015). Another study on young adults concluded that total physical activity was 1,655 MET min/week, which they classified as low (Tek et al., 2020). Since we know that the IPAQ-SF tends to underestimate physical activity (Craig et al., 2003), the mean value of our participants can be considered comparatively high.

### Strengths and Limitations

A major strength of the present study is that the baseline data were collected before the COVID-19 pandemic using a validated instrument, which allowed a quantification of the physical activity levels. A limitation is that the sample size is rather small compared to other studies and possible differences would be more obvious in a larger group. All participants were from the German-speaking region of Switzerland, had a very high educational level and were mostly Swiss nationals, resulting in comparatively high MET min/week values being reported. They were employed by the local government and were working mainly from home at the time of the lockdown, so there was no reduction in working hours, and employment level (full-time vs. part-time) did not change. Thus, our results cannot be generalized to all (office) workers, especially not to those in the private sector, where the COVID-19 pandemic may have led to substantial changes in work organization and increased unemployment (e.g., more leisure time).

Regarding the measurement tool IPAQ-SF, the recall bias seems to be rather low compared to other questionnaires with only 7 days (Sember et al., 2020). However, the IPAQ-SF does not distinguish between different domains of physical activity (i.e., occupational, domestic, transportation, and leisure time), and these usually provide different effects on health. In addition, the IPAQ-SF is known to underestimate the true values of physical activity (Lee et al., 2011) and no minimal clinically important difference in MET min/week was declared, which would be necessary for the correct interpretation of the results.

As all data were collected using an online questionnaire, social desirability bias and response bias cannot be excluded (subjectivity). Participants completed the IPAQ-SF for the first time in January, which may have resulted in a higher questionnaire bias of the baseline data. The follow-up in April included some holidays, so these values may be less representative for this time point (more leisure time).

### Implementation

To remain physically and mentally healthy during the ongoing COVID-19 pandemic, it is important to follow the recommendations for minimal physical activity while respecting the rules of social distance. In view of a possible further lockdown in the coming months, the authorities and government should educate (Chtourou et al., 2020), inform, and raise awareness of the need for sufficient physical activity. Lockdown induced reduction of physical activity can be compensated by individuals by increased leisure-time activity.

### Further Research

Further research in larger study populations is warranted to investigate physical activity more closely, especially with the use of an objective measurement tool, where a minimal clinically important difference is known. It would also have to be investigated to what extent the season has an influence on the values of physical activity and whether a correction based on the season would be necessary. The comparison of our data with those of other countries would also be relevant, especially when comparing the different governmental restrictions.

## Conclusion

The COVID-19 pandemic forced many countries into a societal-level lockdown. In this study, we investigated the effect of the COVID-19 pandemic on the physical activity among Swiss office workers. The hypothesis of a reduction in total physical activity levels during the lockdown in April 2020 compared to the level before the COVID-19 pandemic became effective in Switzerland (January 2020) was rejected. Before the lockdown phase 71% met the minimum level of recommended total physical activity. During the first weeks of the lock-down 54% of participants maintained their physical activity level and 29% showed an increase.

## Members of the Nexpro Collaboration Group

Andrea M. Aegerter (Switzerland), Marco Barbero (Switzerland), Beatrice Brunner (Switzerland), Jon Cornwall (New Zealand), Yara Da Cruz Pereira (Switzerland), MD (Switzerland), OD (Switzerland), JD (Switzerland), HD (Switzerland), Tobias Egli (Switzerland), AE (Switzerland), Markus J. Ernst (Switzerland), Irene Etzer-Hofer (Switzerland), Deborah Falla (United Kingdom), Michelle Gisler (Switzerland), Michelle Haas (Switzerland), VJ (Australia), Sandro Klaus (Switzerland), Gina M. Kobelt (Switzerland), Kerstin Lüdtke (Germany), HL (Switzerland), MM (project leader, Switzerland), Corinne Nicoletti (Switzerland), Seraina Niggli (Switzerland), Achim Nüssle (Switzerland), Salome Richard (Switzerland), Nadine Sax (Switzerland), Katja Schülke (Switzerland), GS (Denmark), Lukas Staub (Australia), TV (Switzerland), and Thomas Zweig (Switzerland).

## Data Availability Statement

The raw data supporting the conclusions of this article will be made available by the authors, without undue reservation.

## Ethics Statement

The studies involving human participants were reviewed and approved by the Ethical Commission of the Canton of Zurich, Switzerland (15.10.2019, swissethics No. 2019-01678). The patients/participants provided their written informed consent to participate in this study.

## Author Contributions

AMA and MD wrote the manuscript. MD, AMA, and TV performed the statistical analysis. GS, VJ, TV, HL, JD, HD, OD, MM, and AE revised the manuscript. All authors read and approved the final manuscript.

## Conflict of Interest

The authors declare that the research was conducted in the absence of any commercial or financial relationships that could be construed as a potential conflict of interest.
