# Supplementary figures and images for: No Evidence for a Decrease in Physical Activity Among Swiss Office Workers During COVID-19: A Longitudinal Study
Source: Front Psychol. 2021 Feb 11;12:620307. doi: 10.3389/fpsyg.2021.620307 (PMC7928288; doi:10.3389/fpsyg.2021.620307)

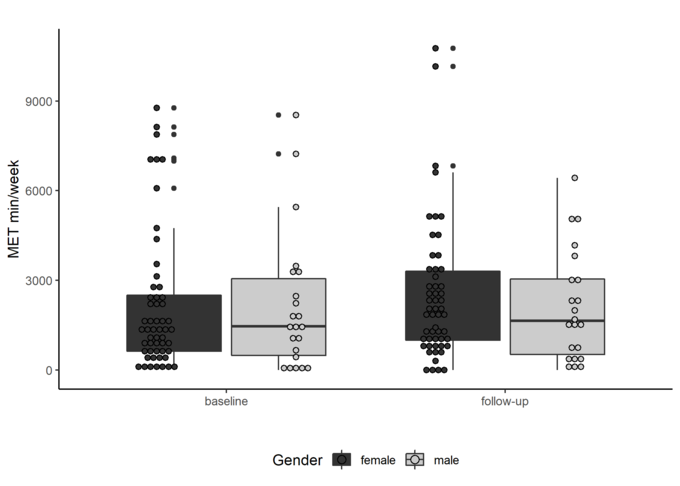

Supplement: Supplementary file 1 [file Image_1.TIFF]

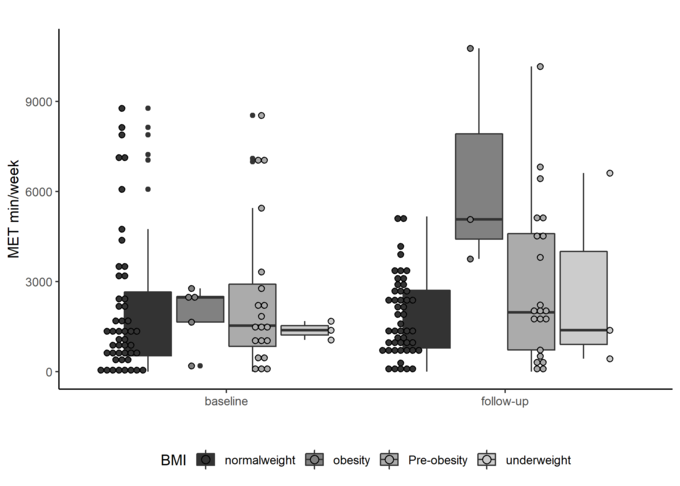

Supplement: Supplementary file 2 [file Image_2.TIFF]
