# Supplementary material for: No Evidence for a Decrease in Physical Activity Among Swiss Office Workers During COVID-19: A Longitudinal Study
Source: Front Psychol. 2021 Feb 11;12:620307. doi: 10.3389/fpsyg.2021.620307 (PMC7928288; doi:10.3389/fpsyg.2021.620307)
Supplement: Supplementary file 4 [file Presentation_1.pdf]

Supplementary Material

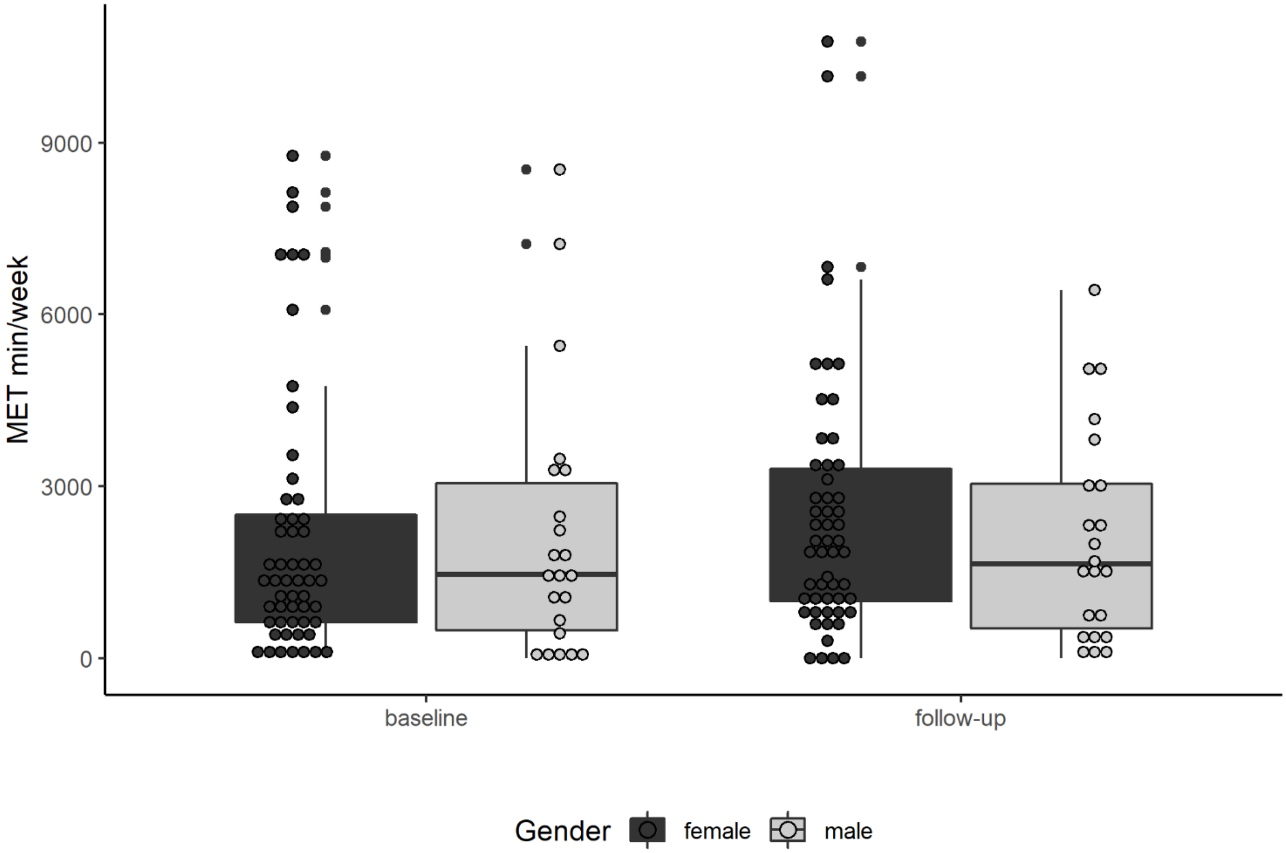

Figure 4: Physical activity in MET minutes/week at baseline (before the COVID-19 pandemic) and follow-up (during the lockdown), stratified by gender

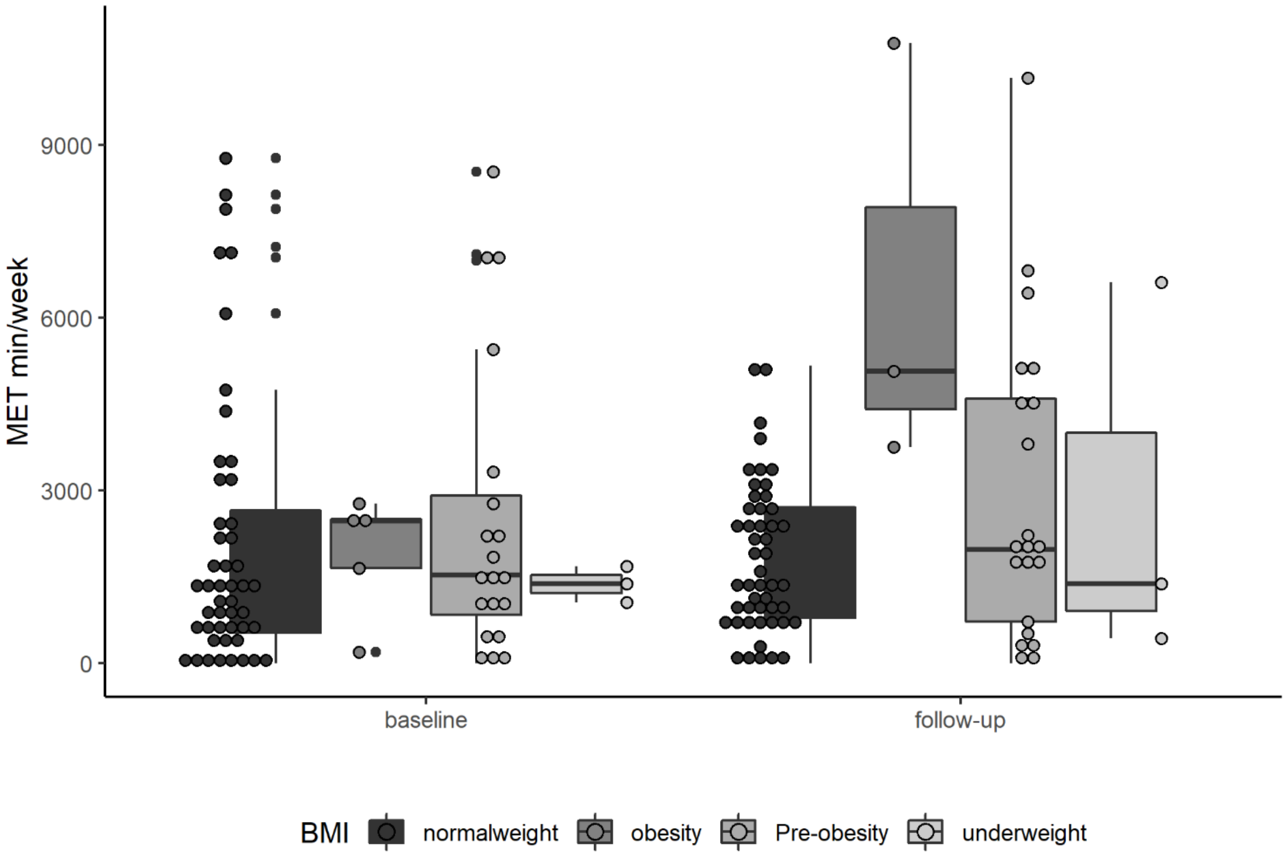

Figure 5: Physical activity in MET minutes/week at baseline (before the COVID-19 pandemic) and follow-up (during the lockdown), stratified by body-mass-index according to World Health Organization classification
